# Morphology and genomic hallmarks of breast tumours developed by *ATM* deleterious variant carriers

**DOI:** 10.1186/s13058-018-0951-9

**Published:** 2018-04-17

**Authors:** Anne-Laure Renault, Noura Mebirouk, Laetitia Fuhrmann, Guillaume Bataillon, Eve Cavaciuti, Dorothée Le Gal, Elodie Girard, Tatiana Popova, Philippe La Rosa, Juana Beauvallet, Séverine Eon-Marchais, Marie-Gabrielle Dondon, Catherine Dubois d’Enghien, Anthony Laugé, Walid Chemlali, Virginie Raynal, Martine Labbé, Ivan Bièche, Sylvain Baulande, Jacques-Olivier Bay, Pascaline Berthet, Olivier Caron, Bruno Buecher, Laurence Faivre, Marc Fresnay, Marion Gauthier-Villars, Paul Gesta, Nicolas Janin, Sophie Lejeune, Christine Maugard, Sébastien Moutton, Laurence Venat-Bouvet, Hélène Zattara, Jean-Pierre Fricker, Laurence Gladieff, Isabelle Coupier, CoF-AT, GENESIS, kConFab, Georgia Chenevix-Trench, Janet Hall, Anne Vincent-Salomon, Dominique Stoppa-Lyonnet, Nadine Andrieu, Fabienne Lesueur

**Affiliations:** 1 INSERM, U900, Paris, France; 2 Institut Curie, Paris, France; 3 Mines Paris Tech, Fontainebleau, France; 4 PSL Research University, Paris, France; 5 Service de Pathologie, Institut Curie, Paris, France; 6 INSERM U830, Paris, France; 7 Service de Génétique, Institut Curie, Paris, France; 8 Unité de Pharmacogénomique, Institut Curie, Paris, France; 9 Institut Curie Genomics of Excellence (ICGex) Platform, Institut Curie, Paris, France; 10 CHU Estaing, CHU Clermont-Ferrand, Clermont-Ferrand, France; 11 Unité de Pathologie Gynécologique, Centre François Baclesse, Caen, France; 12 Service d’Oncologie Génétique, Gustave Roussy, Villejuif, France; 13 Institut GIMI, CHU de Dijon, Hôpital d’Enfants, Dijon, France; 14 Oncogénétique, Centre de Lutte contre le Cancer Georges François Leclerc, Dijon, France; 15 Département d’Hématologie et d’Oncologie Médicale, CLCC Antoine Lacassagne, Nice, France; 16 Service d’Oncogénétique Régional Poitou-Charentes, Centre Hospitalier Georges-Renon, Niort, France; 17 Service de Génétique, Clinique Universitaire Saint-Luc, Brussels, Belgium; 18 Service de Génétique Clinique Guy Fontaine, Hôpital Jeanne de Flandre, Lille, France; 19 Laboratoire de Diagnostic Génétique, UF1422 Oncogénétique Moléculaire, Hôpitaux Universitaires de Strasbourg, Strasbourg, France; 20 Oncogénétique Evaluation familiale et suivi, UF6948 Oncogénétique, Hôpitaux Universitaires de Strasbourg, Strasbourg, France; 21 Laboratoire Maladies Rares: Génétique et Métabolisme, CHU de Bordeaux-GH Pellegrin, Bordeaux, France; 22 Service d’Oncologie Médicale, Hôpital Universitaire Dupuytren, Limoges, France; 23 Département de Génétique, Hôpital de la Timone, Marseille, France; 24 Unité d’Oncogénétique, Centre Paul Strauss, Strasbourg, France; 25 IUCT Oncopole, Institut Claudius Regaud, Toulouse, France; 26 Service de Génétique Médicale et Oncogénétique, Hôpital Arnaud de Villeneuve, CHU de Montpellier, Montpellier, France; 27 Unité d’Oncogénétique, ICM Val d’Aurelle, Montpellier, France; 28 Research Department, Peter MacCallum Cancer Centre, Melbourne, Australia; 29 The Sir Peter MacCallum Department of Oncology, University of Melbourne, Parkville, Australia; 30 Department of Genetics and Computational Biology, QIMR Berghofer Medical Research Institute, Brisbane, Australia; 31 UMR INSERM 1052, Lyon, France; 32 CNRS 5286, Lyon, France; 33 Centre de Recherche en Cancérologie de Lyon, Lyon, France; 34 Université Paris Descartes, Paris, France

**Keywords:** *ATM*, Breast tumour, Pathology, Genetic instability, OncoScan array, Copy number, Loss of heterozygosity, Genomic signature

## Abstract

**Background:**

The ataxia telangiectasia mutated (*ATM*) gene is a moderate-risk breast cancer susceptibility gene; germline loss-of-function variants are found in up to 3% of hereditary breast and ovarian cancer (HBOC) families who undergo genetic testing. So far, no clear histopathological and molecular features of breast tumours occurring in *ATM* deleterious variant carriers have been described, but identification of an *ATM*-associated tumour signature may help in patient management.

**Methods:**

To characterise hallmarks of *ATM*-associated tumours, we performed systematic pathology review of tumours from 21 participants from ataxia-telangiectasia families and 18 participants from HBOC families, as well as copy number profiling on a subset of 23 tumours. Morphology of *ATM*-associated tumours was compared with that of 599 patients with no *BRCA1* and *BRCA2* mutations from a hospital-based series, as well as with data from The Cancer Genome Atlas. Absolute copy number and loss of heterozygosity (LOH) profiles were obtained from the OncoScan SNP array. In addition, we performed whole-genome sequencing on four tumours from *ATM* loss-of-function variant carriers with available frozen material.

**Results:**

We found that *ATM*-associated tumours belong mostly to the luminal B subtype, are tetraploid and show LOH at the *ATM* locus at 11q22–23. Unlike tumours in which *BRCA1* or *BRCA2* is inactivated, tumours arising in *ATM* deleterious variant carriers are not associated with increased large-scale genomic instability as measured by the large-scale state transitions signature. Losses at 13q14.11-q14.3, 17p13.2-p12, 21p11.2-p11.1 and 22q11.23 were observed. Somatic alterations at these loci may therefore represent biomarkers for *ATM* testing and harbour driver mutations in potentially ‘druggable’ genes that would allow patients to be directed towards tailored therapeutic strategies.

**Conclusions:**

Although *ATM* is involved in the DNA damage response, *ATM*-associated tumours are distinct from *BRCA1*-associated tumours in terms of morphological characteristics and genomic alterations, and they are also distinguishable from sporadic breast tumours, thus opening up the possibility to identify *ATM* variant carriers outside the ataxia-telangiectasia disorder and direct them towards effective cancer risk management and therapeutic strategies.

**Electronic supplementary material:**

The online version of this article (10.1186/s13058-018-0951-9) contains supplementary material, which is available to authorized users.

## Background

Ataxia-telangiectasia (A-T) is a rare autosomal recessive disorder caused by biallelic inactivating variants in the *ataxia-telangiectasia mutated* (*ATM*) gene. The phenotype is characterised by progressive neuronal degeneration, immunological deficiency, genetic instability, hypersensitivity to ionising radiation and agents that cause DNA double-strand breaks, and a predisposition to malignancies, particularly lymphoid tumours [1–3]. Epidemiological studies on A-T families showed that heterozygous *ATM* deleterious variant carriers (hereafter referred to as HetAT) are also at increased risk of other cancer types [4–6], notably of breast cancer (BC) in female relatives [7, 8]. It is estimated that 0.5% to 1% of the general population are HetAT, and studies conducted in hereditary breast and ovarian cancer (HBOC) families or early-onset BC cases showed that deleterious *ATM* alleles confer a two- to four-fold increase in BC risk for carriers as compared with non-carriers [9, 10]. Therefore, most published case-control studies or family-based studies described such *ATM* alleles as moderate-risk BC susceptibility alleles, although this risk may differ according to the type of variant [9, 10]. Consequently, *ATM* is now included in nearly all multigene panels used for HBOC genetic testing that include, in addition to *BRCA1* and *BRCA2,* other moderate- to high-risk genes coding for tumour suppressor proteins acting in critical processes of DNA repair pathways [11]. However, results of *ATM* testing are usually not issued to patients, owing to the imprecise absolute risk estimates and a lack of management recommendations for *ATM* variant carriers and their relatives [11]. Nevertheless, National Comprehensive Cancer Network guidelines recommend an annual screening mammogram and annual MRI with contrast enhancement beginning at age 40, or earlier based on family BC history for HetAT women [12]. Moreover, Australia has national best practice guidelines addressing the variant c.7271T>G; these guidelines are based on those that apply to the management of BC risk in *BRCA2* deleterious variant carriers [13, 14]. If a consensus was made to define *ATM* as a gene with clinical utility, specific pathological and genomic features associated with *ATM* inactivation in tumours could help identify subjects with no strong personal or family history of BC. A genetic test for *ATM* may thus be offered to them and their relatives and thereby direct those individuals towards effective cancer risk management and therapeutic strategies.

So far, no clear histopathological and molecular features have been described for *ATM*-associated breast tumours (*i.e.,* tumours developed by subjects carrying one or two mutated copies of *ATM*), and well-documented *ATM* tumour series are very limited. Researchers in three Australian studies on familial BC investigated breast tumours developed by HetAT participants carrying either a truncating variant (TV) or a missense variant (essentially the c.7271T>G; p.Val2424Gly variant) [13, 15]. The first two studies, carried out on 21 and 35 tumours, respectively, suggested that histologically, breast tumours from HetAT subjects do not resemble the tumours from *BRCA1* mutation carriers [15], and no difference was observed between the histological grade of *ATM-*associated tumours and a series of age-matched control tumours [13]. The third study, focusing on six tumours from carriers of the c.7271T>G variant, revealed that all tumours presented with the luminal A or B molecular subtype [16]. Finally, no consistent pattern of loss of the normal allele was reported in an Australian series (*N* = 17) or in a French series (*N* = 16) of tumours from carriers of putative BC-associated *ATM* variants [13, 17].

The purpose of the present study was to determine whether breast tumours developed by *ATM* variant carriers show distinctive histopathological and genomic features as compared with ‘sporadic’ tumours, and also whether they resemble the breast tumours described in carriers of a deleterious variant of other known BC susceptibility genes, in particular the *BRCA1*-associated phenotype [18]. To this end, we conducted a pathology review of a series of tumours composed of 3 breast tumours from 3 A-T subjects (who were therefore homozygous or compound heterozygous *ATM* variant carriers), 20 tumours from 18 HetAT subjects from A-T families, and 18 tumours from 18 HetAT subjects from HBOC families who were non-carriers of other known high-risk variants. We also performed single-nucleotide polymorphism (SNP) array genomic profiling to assess somatic loss of heterozygosity (LOH) at the *ATM* locus and to investigate absolute copy number and LOH profiles at the genome-wide level. Finally, to complete the repertoire of somatic alterations of *ATM*-associated breast tumours, we performed whole-genome sequencing (WGS) on four tumours from HetAT participants with available frozen material.

## Methods

### Study participants and tumour material

Breast tumour samples were selected from carriers of a deleterious *ATM* variant from four different research resources: the French retrospective study on A-T families (Retro-AT) [19], the French prospective cohort on women related to an A-T child (CoF-AT) [8], the GENESIS study [20] and the Kathleen Cuningham Foundation Consortium for research into Familial Breast Cancer (kConFab) study [21]. Briefly, Retro-AT [19] was carried out between 1994 and 1997 to assess cancer risk in A-T families living in France. Thirty-four A-T families were identified during this period, and 27 of them were subsequently included in the CoF-AT prospective study. CoF-AT is an ongoing national prospective cohort study of A-T families which was initiated in 2003 to investigate environmental and genetic risk factors for BC in HetAT and non-HetAT (*i.e.,* non-carriers of an *ATM* variant) women. All women aged 18 and over were eligible to participate in the study. At inclusion, participants provided a blood sample to determine whether they carried one of the *ATM*-inactivating variants identified in the A-T child of the family. As of June 2017, 415 women (213 HetAT and 202 non-HetAT) belonging to 105 A-T families had been enrolled in CoF-AT, and 37 study participants from Retro-AT or CoF-AT had developed BC, including 23 HetAT women, 11 non-HetAT women and 3 A-T subjects having inherited two inactivated copies of *ATM* (2 females and 1 male). Breast tumour material from the 3 A-T subjects and from 18 HetAT subjects could be retrieved for the present study (Table 1).

**Table 1:** Clinical characteristics of *ATM* mutation carriers, and available tumour material used for analyses

Study	Patient ID	Sex	Nucleotide change	Effect on protein	Variant type*	Tumour ID	Age at diagnosis	Stade	Neoadjuvant treatment	Conservation	OncoScan Analysis
A-T families (Retro-AT + CoF-AT)	AT1	M	c.2839-580_577del4(-/-)	cryptic splice site	TV ^(1)^	T0072-L	28	Invasive	Unknown	FFPE	Yes
	AT2	F	c.8585-2A>C	frameshift	TV	T0075-L	42	Invasive	Unknown	Bouin	No
			c.5189G>T	p.Arg1730Leu	MS (C0)						
	AT3	F	c.2413C>T	p.Arg805X	TV	T0249-L	30	Invasive	Unknown	FFPE	Yes
			c.7517_7520delGAGA	p.Arg2506ThrfsX3	TV						
	1	F	c.3576G>A	p.Ser1135_Lys1192del58	TV	T0001-L	62	Invasive	No	FFPE+Frozen	No
	2	F	c.2839-580_577del4	cryptic splice site	TV	T0002-L	60	Invasive	No	Bouin	No
	3	F	c.5644C>T	p.Arg1882X	TV	T0003-L	45	Invasive	No	FFPE	No
	4	F	c.3802delG	p.Val1268X	TV	T0005-L	65	Invasive	No	FFPE	Yes
	5	F	c.6007-2A>T	frameshift	TV	T0007-R	74	Invasive	No	FFPE	No
	6	F	c.3085dupA	p.Thr1029AsnfsX19	TV	T0008-R	51	Invasive	Unknown	FFPE	No
	7	F	c.3894dupT	p.Ala1299CysfsX3	TV	T0009-L	36	Invasive	No	FFPE	Yes
	8	F	c.6007-2A>T	frameshift	TV	T0010-L	30	Invasive	Unknown	Bouin	No
	9	F	c.6404_6405insTT	p.Arg2136X	TV	T0015-L	40	Invasive	No	FFPE+Frozen	Yes
	10	F	c.2466fs	del exon19-65	TV	T0016-R	62	Invasive	No	Bouin	No
						T0016-L	72	Invasive	No	FFPE	Yes
	11	F	c.3153+1G>A	frameshift	TV	T0073-L	77	Invasive	Unknown	Bouin	No
	12	F	c.8489T>G	p.Val2830Gly	MS (C65)	T0074-L	62	Invasive	Unknown	Bouin	No
	13	F	c.73-2A>G	frameshift	TV	T0076-R	47	Invasive	No	FFPE	Yes
	14	F	c.3754_3756delTATinsCA	p.Met2918IlefsX21	TV	T0077-L	66	Invasive	No	FFPE+Frozen	Yes
						T0077-R	66	Invasive	No	FFPE+Frozen	Yes
	15	F	c.8140C>T	p.Gln2714X	TV	T0078-L	55	Invasive	No	FFPE	Yes
	16	F	c.5644C>T	p.Arg1882X	TV	T0181-R	39	In situ	Unknown	FFPE	No
	17	F	c.8083G>A	p.Gly2695Ser	MS (C55)	T0247-R	48	Invasive	No	FFPE	Yes
	18	F	c.7928-2A>C	frameshift	TV	T0248-L	35	Invasive	Unknown	FFPE	Yes
GENESIS	19	F	c.2413C>T	p.Arg805X	TV	T0045-R	31	Invasive	No	No material	-
						T0045-R	67	In situ	No	FFPE	Yes
	20	F	c.8584+1G>A	frameshift	TV	T0091-L	51	In situ	No	FFPE	No
	21	F	c.3058dupA	p.Leu1019fs	TV	T0099-L	39	Invasive	No	FFPE	Yes
	22	F	c.5497-2A>C	p.Val1833IlefsX	TV ^(2)^	T0111-L	32	Invasive	No	FFPE	No
	23	F	c.9008A>T	p.Asn3003Ile	MS (C65)	T0118-L	64	Invasive	No	FFPE	No
	24	F	c.1464G>T	p.Trp488Cys	MS (C65)	T0120-R	65	Invasive	No	FFPE	Yes
	25	F	c.5527delC	p.Phe1843fs	TV	T0123-R	74	Invasive	No	FFPE	Yes
	26	F	c.5750G>C	p.Arg1917Thr	MS (C65)	T0191-L	45	Invasive	Unknown	FFPE	No
	27	F	c.1236-2A>T	p.Trp412X	TV	T0192-R	42	In situ	Unknown	FFPE	No
	28	F	c.8614C>A	p.His2872Asn	MS (C65)	T0218-R	54	Invasive	Unknown	FFPE	No
	29	F	c.8494C>T	p.Arg2832Cys	MS ^(3)^ (C45)	T0220-R	42	Invasive	No	FFPE	Yes
kConFab	30	F	c.3801delG	p.Glu1267fs	TV	T0173-L	44	Invasive	Unknown	No material	-
						T0173-R	49	Invasive	Unknown	FFPE	Yes
	31	M	c.6820G>A	p.Ala2274Thr	MS (C55)	T0174-R	45	In situ	Unknown	FFPE	Yes
	32	F	c.4909+1G>A	frameshift	TV ^(4)^	T0175-R	42	Invasive	Unknown	FFPE	Yes
	33	F	c.8266A>T	p.Lys2756X	TV ^(5)^	T0176-R	60	Invasive	Unknown	FFPE	Yes
	34	F	c.8158G>C	p.Asp2720His	MS (C65)	T0177-L	59	Invasive	Unknown	FFPE	No
	35	F	c.8266A>T	p.Lys2756X	TV ^(5)^	T0179-R	41	Invasive	Unknown	No material	-
						T0179-L	50	Invasive	Unknown	FFPE	Yes
	36	F	c.7176_7177insT	p.Ser2394PhefsX9	TV	T0180-R	43	Invasive	Unknown	FFPE	Yes

*AT* ataxia-telangiectasia, *F* female, *M* male, *TV* truncating variant, *MS* missense substitution. *L* left, *R* right, *FFPE* formalin-fixed, paraffin-embedded tissue sample, *Bouin* Bouin-fixed, paraffin-embedded tissue sample

(1,2,3,4,5) Reported as pathogenic for A-T in ClinVar

*Align-GVGD grades are indicated in brackets for MS variants

GENESIS is a national study on HBOC families identified through French family cancer clinics [20]. Index cases were women diagnosed with invasive mammary carcinoma or *in situ* ductal carcinoma, having at least one sister affected with BC, and with a negative test result for a pathogenic variant in *BRCA1* and *BRCA2*. *ATM* carriers of a TV or of a rare likely deleterious missense substitution (MS) were identified during the course of a large-scale case-control mutation-screening study (F. Lesueur, PhD, unpublished data, March 2018). Tumour material from 11 of them was assessed in the present study (Table 1). In addition, we investigated tumours from seven HetAT subjects enrolled in the Australian kConFab study [21] (Table 1).

### Selection of *ATM* variant carriers

Individuals included in the study were either homozygous, compound heterozygous or heterozygous carriers of a variant considered pathogenic for A-T disorder. We also selected HetAT BC participants from HBOC families carrying a TV that had not necessarily been reported in A-T families, as well as carriers of a rare likely deleterious MS classified as C65, C55 or C45 according to the Align-GVGD tool as previously described [10, 22]. Carriers of the p.Val2424Gly variant identified in kConFab were not included in this study, because tumour characteristics of carriers of this variant had been already investigated [13, 15, 16]. In total, 41 tumour tissues were available from 3 A-T and 36 HetAT subjects for histopathological review (Table 1).

### Pathology review

The Hematoxylin and Eosin Stained (HES) breast tumour tissue was reviewed and scored for morphology features and graded by two pathologists (AVS and GB) using the modified system of Elston et al. [23]. The World Health Organisation classification of tumours of the breast was used to determine histological subtype of *ATM*-associated tumours, and TNM stage according to tumour size, nodal infiltration and metastasis status [24]. Oestrogen receptor (ER), progesterone receptor (PR) and human epidermal growth factor receptor 2 (HER2) status, as well as the expression of proliferating marker Ki-67, was obtained from histopathology reports held by diagnostic laboratories. When incomplete, hormonal status was determined by immunohistochemistry (IHC) staining at Institut Curie. Tumours were considered HER2+ if they were scored 3+ by IHC or for tumours scored 2+ by IHC if fluorescence in situ hybridisation showed an *HER2* gene amplification. Tumours were classified using IHC data according to the St. Gallen molecular subtypes as follows: triple-negative (ER−, PR− and HER2−), HER2-overexpressing (ER−, PR− and HER2+), luminal A (ER+, PR+/−, HER2− and Ki-67 < 20%), luminal B (ER+, PR+/−, HER2− and Ki-67 ≥ 20%), and luminal B/HER2+ (ER+, PR+/−, HER2+ and Ki-67 ≥ 20%) [25].

Morphological features of *ATM*-associated breast tumours were compared with the series of breast tumours from patients who had surgery at Institut Curie between 2005 and 2006, named the PICBIM series (from the *programme incitatif et collaboratif - Cancer du sein: invasion et motilité*). None of the PICBIM patients received neoadjuvant treatment. Patients who had developed a previous cancer at any site were excluded, as were known *BRCA1* or *BRCA2* mutation carriers. In this series, *ATM* mutation status of participants had not been determined. In total, 516 patients diagnosed with invasive carcinoma and 83 patients diagnosed with *in situ* carcinoma served as control subjects.

### DNA preparation and confirmation of the familial *ATM* deleterious variant

Tumour DNA was extracted from tumour-enriched areas (with ≥ 50% tumour content when possible) delimited from the most representative HES-stained slides for the 35 tumours for which formalin-fixed, paraffin-embedded (FFPE) material was available (Table 1). The relevant areas were macrodissected from four 10-μm sections, and DNA was purified using the NucleoSpin Tissue protocol according to the manufacturer’s instructions (Macherey-Nagel, Düren, Germany). DNA quantity and quality were assessed using a Qubit fluorometer (Life Technologies/Thermo Fisher Scientific, Carlsbad, CA, USA) and SYBR Green-based qPCR assay (Promega, Madison, WI, USA). Of the 35 available FFPE tumour DNA samples, sufficient quantity and quality to perform subsequent molecular analyses were obtained for 23 of them. Matched blood DNA was extracted with the QuickGene-610L automated system (AutoGen, Holliston, MA, USA) according to the manufacturer’s instructions. The presence of the familial *ATM* deleterious variant was confirmed in all analysable blood and tumour DNA samples by Sanger targeted sequencing on the Applied Biosystems ABI 3500xL DNA analyser (Thermo Fisher Scientific, Forest City, CA, USA).

### Copy number variation analysis

Copy number variation (CNV) analysis using the Affymetrix OncoScan SNP array (Thermo Fisher Scientific, Santa Clara, CA, USA) was performed on the 23 FFPE *ATM*-associated tumours, including 2 tumours from 2 A-T participants (Table 1). Data were analysed with the Genome Alteration Print (GAP) method, which takes into account both ploidy and large-scale genomic rearrangements [18, 26]. Copy number ranged from zero to eight copies, and all segments exceeding eight copies were ascribed eight-copy status. Chromosome number was estimated by the sum of the copy numbers detected at the peri-centric regions. Output processing files derived from the GAP tool were analysed using VAMP in-house software [27] to define the boundaries of regions recurrently altered in *ATM*-mutated tumours. Copy loss and gain for near-diploid tumours were called for the segments with zero or one copy and four or more copies, respectively. Copy loss and gain for near-tetraploid tumours were called for the segments with less than or equal to two and six or more copies, respectively. LOH status was ascribed to regions having monoallelic content, regardless of copy number. LOH associated with copy loss was referred as LOH/loss. Breakpoints (changes in the copy number or major allele counts within chromosomes) in each genomic profile were characterised on the basis of resulting absolute copy number profile and after filtering for regions with < 50 SNP variations. Recurrent alterations (CNV, LOH) among the cohort were obtained using the CNTools R package (version 1.24.0; R Foundation for Statistical Computing, Vienna, Austria) and a homemade script. In order to find inherent grouping structure, hierarchical clustering was performed using the alteration status (absence/presence of an alteration) per segment using the Jaccard distance and the Ward linkage function, available in the vegan R package (version 2.3-3).

### Validation of LOH status at *ATM* locus using microsatellites

Tumour and matched blood DNA were evaluated on a subset of participants by using a PCR-based LOH assay with four fluorescence-labelled microsatellite markers (namely D11S1113, D11S1819, D11S2179 and D11S1778) spanning a 14.4-Mbp region encompassing the *MRE11A* and *ATM* genes. Capillary electrophoresis was performed on the ABI 3500xL DNA analyser. Raw electrophoretic data were analysed with GeneMarker software version 1.3 (SoftGenetics, LLC, State College, PA, USA) to assess allele ratios. We considered LOH at the *ATM* locus when the allele ratio fell below 50% in the tumour DNA sample.

### Whole-genome sequencing

WGS was performed on four tumour-normal DNA pairs from three participants for whom frozen tumour tissue was available (Table 1). Paired-end libraries were prepared from 2 μg of DNA using the TruSeq DNA PCR-Free Low-Throughput Sample Preparation Kit (Illumina, San Diego, CA, USA) and were sequenced on the HiSeq 2500 instrument (Illumina). Tumour DNA was sequenced at a higher depth of coverage (100×) than the germline counterpart (30×). Sequencing reads were mapped to the reference genome (assembly hg19) using Burroughs-Wheeler Aligner (version 0.7.5a) [28]. Regions of CNV and LOH were identified using the FACETS algorithm (version 0.5.6) [29], and single-nucleotide variations (SNVs) and indels were called using VarScan 2 [30]. Somatic variants were filtered and annotated using an in-house pipeline.

### Statistical analysis

Statistical analyses were performed using STATA version 14.1 software (StataCorp, College Station, TX, USA). Two-tailed tests with a 5% significance level were used throughout. Logistic regressions were used to assess the level of association between the presence of an *ATM* variant and various features of interest when comparing the *ATM* series with the PICBIM series. Fisher’s exact test (FET) was used to assess molecular subtype differences between *ATM*-associated tumours and breast tumours from sporadic cases from The Cancer Genome Atlas (TCGA) [31] and from the Norwegian series [32].

## Results

### Histopathological features associated with *ATM* variant status

Clinicopathological and IHC features were evaluated on 3 breast tumours from 3 A-T participants and 38 tumours from 36 HetAT participants. This tumour series was compared with BC cases enrolled in the PICBIM program of Institut Curie. An overview of the features examined in both series is presented in Table 2.

**Table 2:** Clinical and histological features of *ATM*-associated invasive breast carcinomas compared with those of sporadic cases

Clinicopathological variable	PICBIM series (*N* = 516)	*ATM* series (all tumours, TV + MS) (*N* = 36)	*P value* ^a^	*ATM* series (1st primary tumours only) (*N* = 32)	*P value* ^a^	*ATM* series (excluding MS in HBOC families) (*N* = 31)	*P value* ^a^
Histological subtype							
Ductal carcinoma	434	30	Reference	27	Reference	25	Reference
Lobular carcinoma	54	2	0.55	2	0.69	2	0.78
Others	28	3	0.43	2	0.78	3	0.25
Unknown	0	1	–	1	–	1	–
Histological grade							
I	88	4	Reference	3	Reference	4	Reference
II	191	16	0.34	15	0.24	13	0.58
III	236	15	0.64	13	0.55	14	0.75
Unknown	1	1	–	1	–	–	–
Architecture							
1–2	126	0	Reference	7	Reference	6	Reference
3	390	30	0.97	21	0.86	20	0.96
Unknown	0	6	–	4	–	5	–
Mitosis							
0–1	221	15	Reference	14	Reference	12	Reference
2	103	9	0.58	8	0.70	9	0.31
3	191	6	0.10	6	0.13	5	0.15
Unknown	1	6	–	4	–	5	–
Nuclear grade							
1	30	2	Reference	2	Reference	2	Reference
2	225	7	0.25	7	0.22	5	0.12
3	261	21	0.96	19	0.89	19	0.9
Unknown	0	6	–	4	–	5	–
Tumour size (cm)							
pT1 (< 2)	329	23	Reference	20	Reference	19	Reference
pT2 (2–5)	166	8	0.37	7	0.41	7	0.48
pT3 (> 5)	14	2	0.37	2	0.30	2	0.26
pT4	7	0	–	0	–	0	–
Unknown	0	3	–	3	–	3	–
Pushing margins							
Absent	461	19	Reference	17	Reference	15	Reference
Present	49	2	0.98	2	0.93	2	0.78
Unknown	6	15	–	13	–	14	–
Emboli							
Absent	315	13	Reference	11	Reference	13	Reference
Present	199	14	0.08	14	0.04	11	0.32
Unknown	2	9	–	7	–	7	–
N stage							
pN0	286	18	Reference	15	Reference	15	Reference
pN1	151	10	0.80	9	0.66	9	0.55
pN2	59	1	0.23	1	0.32	1	0.35
pN3	16	1	0.96	1	0.81	1	0.76
pNx	5	6	–	6	–	5	–
Oestrogen receptor							
Positive	307	34	Reference	31	Reference	29	Reference
Negative	209	1	0.003	1	0.004	1	0.005
Unknown	0	1	–	–	–	1	–
Progesterone receptor							
Positive	278	26	Reference	23	Reference	23	Reference
Negative	236	8	0.03	8	0.07	6	0.02
Unknown	2	2	–	1	–	2	–
HER2							
Negative	433	25	Reference	23	Reference	21	Reference
Positive	83	6	0.77	5	0.97	6	0.53
Unknown	0	5	–	4	–	4	–
Ki-67							
< 20%	173	11	Reference	10	Reference	8	Reference
≥ 20%	336	18	0.55	17	0.59	17	0.97
Unknown	7	7	–	5	–	6	–
Molecular subtype							
TNBC	142	1	Reference	1	Reference	1	Reference
HER2	66	0	N/A	0	N/A	0	N/A
Luminal A	180	10	0.06	9	0.08	7	0.13
Luminal B	111	13	0.009	12	0.010	12	0.010
Luminal B/HER2	17	4	0.005	4	0.005	4	0.005
Unknown	0	8	–	6	–	7	–

*Abbreviations: ATM*, Ataxia-telangiectasia mutated, *HBOC* Hereditary breast and ovarian cancer, *HER2* Human epidermal growth factor receptor 2, *MS* Missense substitution, *PICBIM* P*rogramme incitatif et collaboratif - Cancer du sein: invasion et motilité* series, *TV* Truncating variant, *TNBC* Triple-negative breast cancer

^a^ *P* value adjusted for sex and for age at diagnosis

Among the 41 reviewed *ATM*-associated breast tumours, 36 were invasive carcinomas and 5 were *in situ* carcinomas. Overall, subjects with invasive carcinoma and subjects with *in situ* carcinoma from the *ATM* series tend to be diagnosed at a younger age than subjects from the PICBIM series (mean age 52.4 *vs.* 56.2 years, *P* = 0.08 for invasive carcinomas; 45.5 *vs.* 54.1 years, *P* = 0.07 for *in situ* carcinomas). This can be explained by the fact that women related to an A-T child or belonging to an HBOC family are more likely to benefit from early detection of the disease owing to their higher risk of developing BC than the general population. We also compared mean age at diagnosis in participants who developed invasive breast carcinoma between the studies, and we observed no difference (CoF-AT/Retro-AT 51.9, GENESIS 55.9, kConFab 50.6, *P* = 0.90).

Invasive breast carcinomas developed by HetAT and A-T participants were mostly ductal carcinomas (86%) with an intermediate to high grade (II–III), which was the same as the distribution of histological types and grades found in the invasive tumours from the PICBIM series. With respect to the IHC of tumours arising in *ATM* variant carriers, 97% of *ATM*-associated tumours were ER+, which was significantly higher than the proportion of ER+ tumours in the PICBIM series (59%, *P* = 0.004). Low to moderate lymphocytic infiltration was observed in *ATM*-associated tumours (data not shown), but this information was not available in the PICBIM, so no comparison could be performed.

**Fig. 1:**
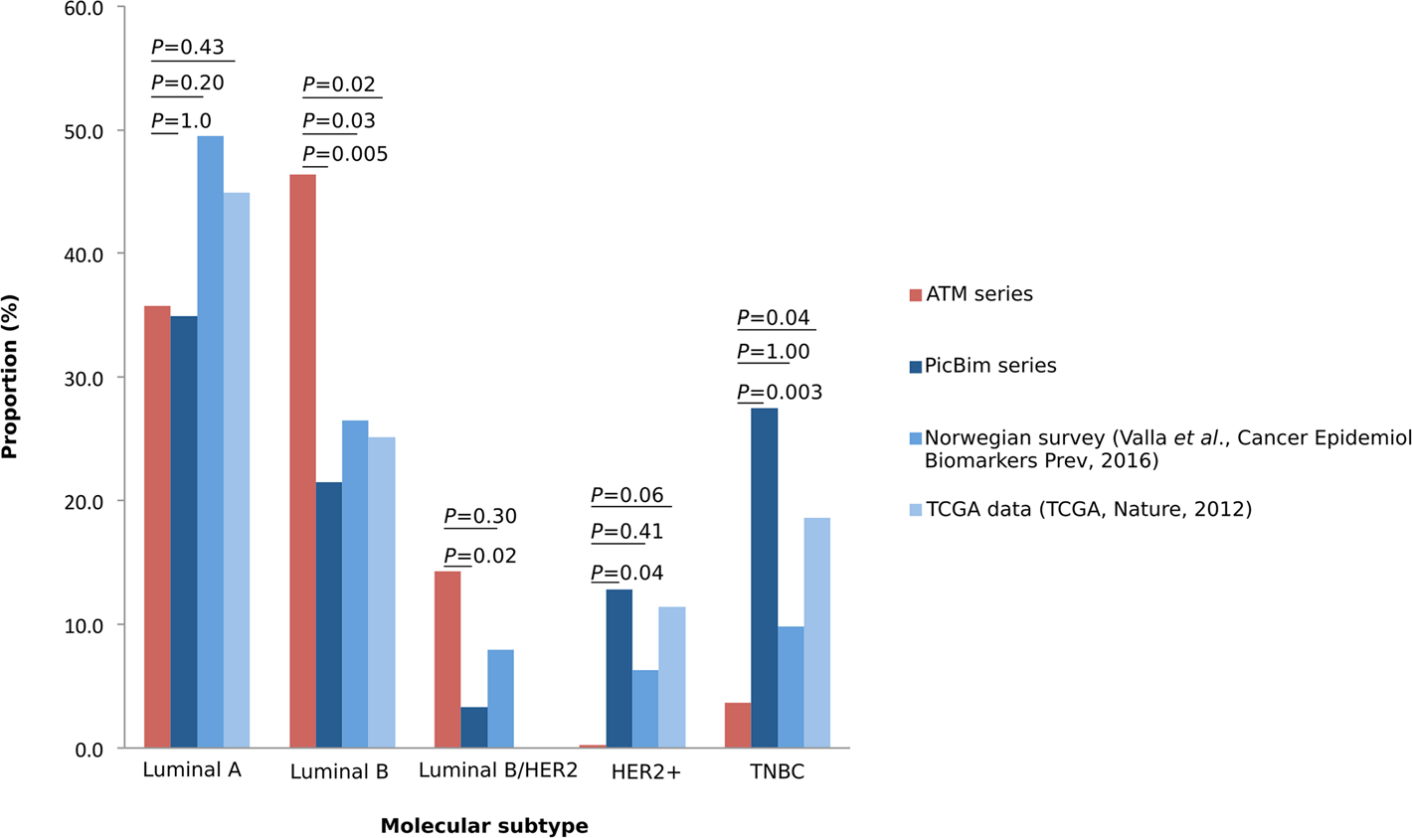
Distribution of molecular subtypes in the ataxia-telangiectasia mutated (*ATM*) series and in the three control series. PAM50 classification was used for The Cancer Genome Atlas (TCGA) data, which explains the absence of luminal B/Human epidermal growth factor receptor 2-positive (HER2+) tumours in this series. Fisher’s exact test was used to assess difference between ATM series and control series. *PICBIM* *Programme incitatif et collaboratif - Cancer du sein: invasion et motilité series*

Molecular subtypes could be determined for 28 of 36 *ATM*-associated invasive breast tumours. *ATM*-associated breast tumours were mostly luminal B (46%) and luminal A (36%), and the distribution of the molecular subtypes differed significantly from that of the PICBIM series. In particular the luminal B and luminal B/HER2+ subtypes were over-represented among tumours developed by HetAT and A-T participants (*P* = 0.009 and *P* = 0.005, respectively) (Table 2). Because the PICBIM series might not reflect the distribution of the molecular subtypes of invasive breast tumours in the general population, we also compared the *ATM* series with a series of 1423 primary breast tumours from a Norwegian population-based survey of women born between 1886 and 1977 [32], as well as with 501 invasive breast tumours characterised with the PAM50 test [33, 34] available in TCGA, after exclusion of carriers of a TV in *BRCA1*, *BRCA2* and *ATM* [31]. We found that the proportion of luminal B tumours was also significantly higher in *ATM*-associated tumours than in the Norwegian study (*P*_FET_ = 0.03) and in the TCGA series (*P*_FET_ = 0.02), whereas the prevalence rates of luminal B/HER2+ tumours and of triple-negative breast tumours in the two latter series were similar to those observed in *ATM*-associated tumours (Fig. 1).

We next performed two sensitivity tests. First, analyses comparing clinical and histological features of *ATM*-associated tumours with those of the PICBIM series were repeated after exclusion of four *ATM-*associated invasive breast tumours, three of which were second primary tumours (T0016-L, T0173-R and T0179-L) whose morphology and histology might have been affected by treatment of the first primary BC. The fourth tumour (T0077-L) was from a patient with synchronous bilateral tumours (T0077-R and T0077-L); we randomly excluded one of the two tumours to take into account only one tumour per patient in the analysis. The results remained unchanged (Table 2).

Second, we repeated the analyses after exclusion of five invasive tumours developed by carriers of a missense variant not reported so far as pathogenic for A-T, namely c.1464G>T (p.Trp488Cys), c.5750G>C (p.Arg1917Thr), c.8158G>C (p.Asp2720His), c.8614C>A (p.His2872Asn) and c.9008A>T (p.Asn3003Ile), to avoid possible misclassification of the deleterious effect of the variant based on *in silico* prediction only. Again, the results remained unchanged (Table 2).

We also compared features of the five *in situ* carcinomas (two ER+, two ER− and one with undetermined ER status) of the *ATM* series with those of the 83 *in situ* carcinomas from the PICBIM series, and we observed no difference in nuclear grade, tumour size and hormonal status between the two groups of tumours. However, owing to the low number of *in situ* carcinomas observed in this series, it was not possible at this stage to draw any conclusions about the characteristics of the *in situ* tumours developed by HetAT participants.

### Genome-wide copy number and LOH profiles of *ATM* breast tumours

**Fig. 2:**
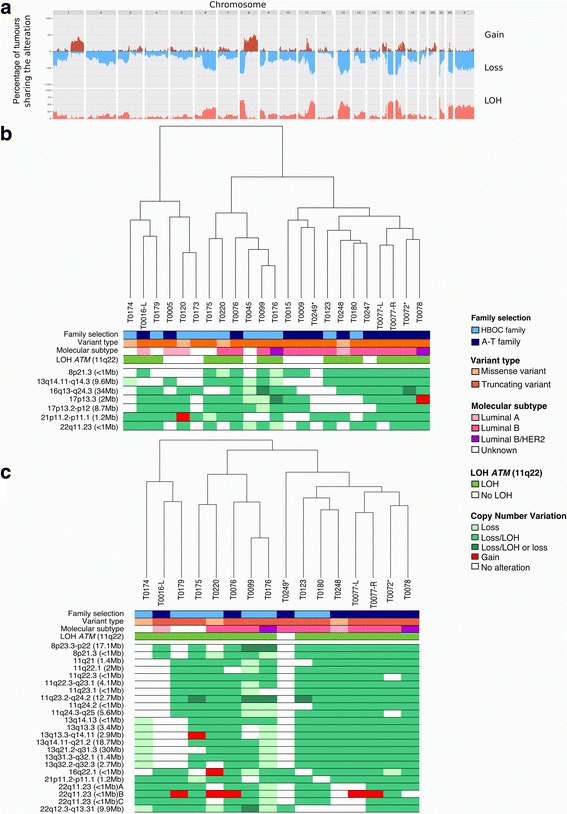
Copy number variation profiles of ataxia-telangiectasia mutated (*ATM)*-associated tumours analysed with the OncoScan array. **a** Genome-wide view of cumulative copy number variations present in the 23 *ATM*-associated tumours. Gains are indicated in *red*, losses in *blue*, and loss of heterozygosity (LOH) in *orange*. **b** Cluster dendrogram and genomic regions altered in ≥ 70% of the 23 analysed tumours. Tumours from Ataxia-telangiectasia (A-T) children are indicated by asterisks. **c** Cluster dendrogram and genomic regions altered in ≥ 70% of the 16 tumours with confirmed biallelic inactivation of *ATM*. Tumours from A-T children are indicated by asterisks. *Loss* The two alleles are present in the tumour, *Loss/LOH* Only one allele is present in the tumour, *Loss/LOH or Loss* Consecutive segmental regions characterised as either ‘Loss/LOH’ or ‘Loss’, *HBOC* Hereditary breast and ovarian cancer, *HER2* Human epidermal growth factor receptor 2

High-quality SNP array data were obtained for 23 FFPE breast tumours; 2 tumours were from A-T subjects, and 21 tumours were from HetAT subjects. Tumour ploidy inferred from the absolute segmental copy number profiles and genotype status by the GAP method [26] identified 16 of 23 (70%) near-tetraploid tumours and 7 of 23 (30%) near-diploid tumours. CNVs and regions of LOH were subsequently determined by taking into account the ploidy of each tumour. No evidence of the homologous recombination deficiency (HRD) signature as measured by large-scale state transition genomic signature [18, 35] was observed among the *ATM*-associated tumours (Fig. 2a).

Because ‘two-hit’ inactivation of the causative gene is regarded as a principal feature of molecular pathogenesis of most hereditary tumours, we next examined the LOH status of *ATM*-associated tumours at 11q22–23 in the tumours from HetAT participants. LOH was found in 14 of the 21 tumours (67%) developed by HetAT participants. Microsatellite analysis performed on a subset of 14 tumours confirmed the OncoScan results, except for 1 tumour (T0005). The one exception may be due to differences in sensitivity of the two methods. In the subsequent analyses, OncoScan results were not considered for this tumour. Sanger sequencing of the tumour-blood DNA pairs suggested that the *ATM* wild-type allele was lost in all tumours that underwent LOH at this locus (data not shown).

Genome-wide profiling of the 23 *ATM*-associated tumours revealed multiple copy number aberrations, including those previously reported in breast tumours, such as losses at 8p and gains at 8q [31], occurring in 50% and 70% of *ATM*-associated tumours, respectively (Fig. 2a and b). Copy number losses at 16q, 17p and 22q, which are known features of breast tumours of the luminal A and B subtypes [31, 36], were also seen in 70% of *ATM*-associated tumours (Fig. 2b). In addition, 70% of *ATM*-associated tumours showed copy number losses at 13q14.11-q14.3, 17p13.2-p12 and 21p11.2-p11.1 (Fig. 2b). The 13q14.11-q14.3 locus is 9.6 Mbp long and contains 90 genes, including *LCP1* (*lymphocyte cytosolic protein 1*) and *RB1* (*RB transcriptional co-repressor 1*) (Table 3)*.* This region is included in the 13q12.3-q21 locus identified in high-grade luminal *BRCA2*-associated tumours as described by Pecuchet et al. [37]. The 17p13.2-p12 locus contains 166 cancer-related genes, including *TP53* and *MAP2K4* (Table 3). The 21p11.2-p11.1 locus is 1.2 Mbp long and contains only the PTEN-related tyrosine phosphatase gene *TPTE,* the pseudogene *TEKT4P2* and four microRNAs (MIR3648-1, MIR3648-2, MIR3687-1 and MIR3687-2) (Fig. 2b and Table 3). A complete list of genes located in segment losses observed in ≥ 70% of *ATM*-associated breast tumours is provided in Additional file 1: Table S1.

**Table 3:** Copy number losses recurrently observed in the 23 *ATM*-associated breast tumours

Locus	Associated morphology	All *ATM* tumours (*N* = 23)			*ATM* tumours with proven biallelic inactivation of *ATM* (*n* = 16)			*ATM* tumours sequenced by WGS (*n* = 4)		
		Cytogenetic band	Number of genes	Cancer genes^a^	Cytogenetic band	Number of genes	Cancer genes^a^	Cytogenetic band	Number of genes	Cancer genes^a^
6q	20% of *ATM* tumours							6q23.3-q27	190	*ARID1B, ECT2L, ESR1, EZR, FGFR10P, QKI, TNFAIP3*
8p	Breast cancer	8p21.3	6^b^	*–*	8p23.3-p12	216	*WRN, NRG1*	8p21.3	6^b^	–
11q	*ATM* tumours	11q22	2	*ATM, ZBTB16*	11q21-q25	276	*ATM, ZBTB16, DDX10, POU2AF1, SDHD, ZBTB16, PAFAH1B2, PDCK7, KMT2A, MAML2, DDX6, BCL9L, CBL, ARHGEF12, KCNJ5*	11q21-q24.2	236	*ATM, ZBTB16, DDX10, POU2AF1, SDHD, ZBTB16, PAFAH1B2, PDCK7, KMT2A, MAML2, DDX6, BCL9L, CBL*
13q	70% *ATM* tumours	13q14.11-q14.3	90	*LCP1, RB1*	13q13.3-q32.3	320	*LHFP, FOXO1, LCP1, RB1*	13q13.3-q32.3	320	*LHFP, FOXO1, LCP1, RB1*
16q	Luminal tumours	16q13-q24.3	364	*HERPUD1, CDH11, CBFB, CTCF, CDH1, ZFHX3, MAF, CBFA2T3, FANCA*	16q22.1	1^c^	*–*	16q22.1	1^c^	–
17p	Luminal tumours	17p13.3	46	*YWHAE*		–	–	17p13.3	46	*YWHAE*
17p	Luminal tumours	17p13.2-p12	166	*USP6, RABEP1, TP53, GAS7, MAP2K4*		–	–			
19p	40% of *ATM* tumours							19p13.3-p13.2	256	*FSTL3, GNA11, MAP2K2, MLLT1, SH3GL1, STK11, TCF3*
21p	70% of *ATM* tumours	21p11.2-p11.1	2^a^	–	21p11.2-p11.1	2^d^	–			
22q	Luminal B tumours, 70% *ATM* tumours	22q11.23	2^b^		22q11.23	3^e^	*GSTT1*			
	Luminal B tumours, 70% *ATM* tumours	–	–	–	22q12.3	199	*APOBEC3B, MKL1, EP300*			

^a^As reported in the COSMIC database

^b^This region contains *PPP3CC*, *SORBS3*, *PDLIM2*, *BIN3*, *BIN3-IT1* and *EGR3*

^c^This region contains *WWP2* and the microRNA MIR140

^d^This region contains *TEKT4P2*, *TPTE* and four miRNAs (MIR3648-1, MIR3648-2, MIR3687-1, MIR3687-2)

^e^This region contains the pseudogenes *GSTTP1* and *GSTTP2*

When we restricted the analysis to the 16 tumours in which biallelic inactivation of *ATM* was demonstrated (*i.e.*, breast tumours from A-T participants and breast tumours from HetAT participants showing LOH at the *ATM* locus), we found that copy number losses at 8p, 11q, 13q and 22q corresponded to longer chromosome segments than the ones described in the 23 *ATM*-associated tumours. The segment loss at 21p11 was the same as the one initially described (Fig. 2c and Table 3).

We also performed unsupervised hierarchical clustering analyses of CNV data. This analysis did not allow separation of *ATM*-associated tumours according to molecular subtype, LOH status at 11q22 (*ATM* locus), type of inherited variant (TV vs. MS) or origin of HetAT participants (A-T families or HBOC families) (Fig. 2b). Interestingly, the synchronous bilateral tumours from HetAT patient T0077 showed similar CNV profiles, whereas tumours from the two A-T participants showed quite distinctive features (Fig. 2b and c).

Finally, although the hierarchical clustering of the CNV data did not separate the tumours according to the variant type (TV vs. MS), we performed a sensitivity analysis excluding tumours of the four HetAT participants carrying an MS. This analysis confirmed that loci 8p21, 13q14, 16q13-q24, 17p13-p12 and 21p11 were sites of recurrent alterations found in ≥ 70% of *ATM*-associated tumours (Additional file 2: Figure S1). However, after exclusion of these 4 tumours, the boundaries of altered loci were extended, and locus 22q11 was lost in only 12 of the 19 analysed tumours.

### Comparison with publicly available data

We used the publicly available data from TCGA [31] accessible through cBioportal [38] to investigate whether the genes listed in Table 3 were specifically lost in *ATM*-associated tumours. A total of 745 TCGA tumour samples with available CNV data were used. Those tumours were from individuals who developed invasive primary breast tumours and did not carry a deleterious variant in *ATM*, *BRCA1* or *BRCA2*. In addition to LOH at the *ATM* locus, which was observed more frequently in *ATM*-associated tumours (67%) than in the TCGA ‘sporadic’ tumours (40.1%) (*P* = 0.02), several genes at other loci appeared more frequently lost in *ATM*-associated tumours, including *TPTE* (21p11.2-p11.1), *GSTT1*, *GSTTP1* and *GSTTP2* (22q11.23), as well as *LCP1, RB1* (13q14), *YWHAE, USP6, RABEP1* and *MAP2K4* (17p13.3-p12) (Additional file 3: Table S2).

### Deep whole-genome sequencing of *ATM*-associated tumours

**Fig. 3:**
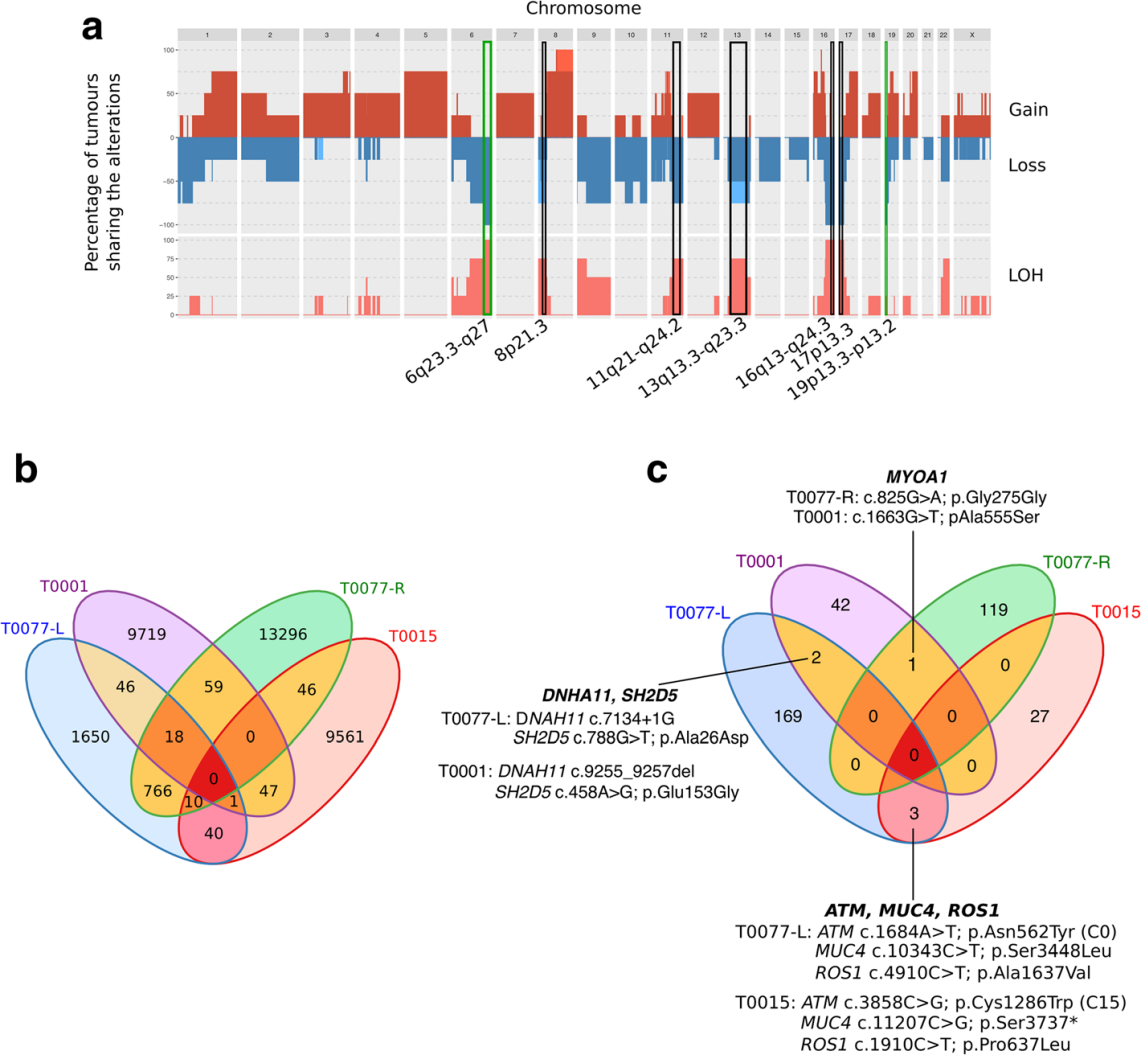
Copy number variation and single-nucleotide variant (SNV) profiles of ataxia-telangiectasia mutated (*ATM*)-associated tumours obtained by deep whole-genome sequencing (WGS). **a** Cumulative profiles of copy number gains, losses and of loss of heterozygosity (LOH) regions obtained from WGS of four *ATM*-associated tumours. *Black boxes* indicate the genomic regions identified in the OncoScan analysis; *green boxes* indicate the new genomic regions identified by WGS. **b** Venn diagram representing the number of somatic SNVs and indels shared between the four tumours. **c** Venn diagram representing the number of genes altered and shared between the four tumours

Whole-genome massively parallel sequencing of four *ATM-*associated breast tumours (T0001-L, T0015-L, T0077-L and T0077-R) and their respective germline DNA was used to characterise the genetic landscape of *ATM*-associated tumours at base pair resolution. Tumour DNA was sequenced at a mean depth of coverage of 97× (range 82×–104×), and paired blood DNA was sequenced at a mean depth of coverage of 36× (range 35×–37×). CNV patterns obtained from WGS data for frozen tumours T0015-L, T0077-L and T0077-R were compared with CNV patterns obtained in the OncoScan analysis in the corresponding FFPE tumours. Loss/LOH was confirmed by whole-genome analysis for loci 8p21.3, 11q21-q24.2 (containing *ATM*), 13q13.3-q32.3, 16q22.1 and 17p13.3, whereas discordant results were obtained at locus 21p11.2-p11.1 for one tumour and at locus 22p11.23 for two tumours (Fig. 3a). Divergent ploidy estimations between the WGS analysis (ploidy 3.5) and the OncoScan analysis (ploidy 4) or the use of different tumour sections to prepare tumour DNA may explain these discrepancies. No OncoScan data were available for tumour T0001, but the CNV profile obtained from the WGS data showed LOH at 11q21-q24.2 (containing *ATM*) and also loss/LOH at 13q13.3-q32.3, 17p13.3 and 22q12.3-q13.31.

In addition we found that the four tumour genomes shared a region of copy number loss/LOH at 6q23.3-q27, which contains *ESR1* encoding the ER, as well as a region of copy number loss at 19p13.3-p13.2 measuring 7.9 Mbp (Fig. 3a) and containing 256 genes (Table 3). Going back to the OncoScan data, we found that these two latter regions were indeed altered but in < 40% of the analysed FFPE tumour genomes.

On the basis of our analysis of high-confidence SNVs identified in each *ATM*-associated tumour genome, we next looked for potential driver mutations. Post-filtering, 51,161 SNVs were identified, 1004 of which were shared by 2 tumours and 29 of which were shared by 3 tumours (Fig. 3b). Only 794 SNVs were shared by the synchronous bilateral tumours T0077-L and T0077-R (Fig. 3b). When analyses were restricted to the coding part of the genome (exome), no genes were found to be altered either in all four tumours or in the two tumours from patient T0077 (Fig. 3c). Six genes were found to be altered in two tumours: *MYO1A*, *DNAH11*, *SH2D5, ATM*, *MUC4* and *ROS1* (Fig. 3c). However, only *DNAH11* variants (c.7134+1G>A and c.9255_9257del) and the nonsense variant in *MUC4* (c.11207C>G) are likely to have a deleterious impact on the gene product function and therefore might represent candidate driver mutations. The two *ATM* somatic variants identified in tumours T0015 and T0077-L were predicted as benign variants according to the Align-GVGD prediction tool (Fig. 3c).

## Discussion

This exploratory study in which we investigated both the histological and molecular features of breast tumours developed by subjects who inherited one or two mutated copies of *ATM* describes, to our knowledge, the largest series of *ATM*-associated tumours reported to date. One asset of the study design is that the vast majority of participants included in the study carried a loss-of-function or missense variant that had been identified in an A-T family, hence avoiding introduction of noise into the analysis that would be due to misclassification of an *ATM* variant based on the impact on the protein function. Moreover, all *ATM*-associated breast tumours and the control series were blindly reviewed by trained reference pathologists of Institut Curie (AVS and GB), thus ensuring unbiased scoring of the morphological features.

The study revealed that most *ATM-*associated breast tumours are luminal B or luminal B/HER2+ tumours, which is consistent with a recent case-control study showing that *ATM* TV carriers are at increased risk of developing ER+ breast tumours [39]. Moreover, tetraploidy, loss of the wild-type allele at the *ATM* locus, and copy number loss/LOH at loci 13q14.11-q14.3, 17p13.2-p12, 21p11.2-p11.1 and 22q11.23 are hallmarks of breast tumours developed by *ATM* variant carriers.

In comparison with breast tumours associated with other BC susceptibility genes, we thus confirm previous observations showing that *ATM*-associated tumours do not resemble *BRCA1-*associated tumours [13, 15] or *PALB2-*associated tumours, which are also predominantly triple-negative tumours [40, 41]. Like *BRCA2-* and *CHEK2-*associated tumours, *ATM-*associated tumours are mostly luminal tumours [42–44] but they do not show a particular histological subtype as observed in *BRCA1-* (medullary) [45], *BRCA2-* (lobular) [45], *CDH1-* (lobular) [46], and *PTEN-*associated tumours (apocrine) [47].

The absence of histological resemblance between *BRCA1-* and *ATM*-associated tumours was reflected at the molecular level. Indeed, *ATM*-associated tumours do not show the HRD signature characterised by large-scale state transitions [18, 48], suggesting that tumorigenesis in *BRCA1* variant carriers and *ATM* variant carriers occurs by different mechanisms. *ATM*-associated tumours also differ from luminal *BRCA2-*associated tumours, which can also display the HRD signature [35]. Our results are consistent with recently published results on tumour-derived genome sequences from seven BCs from TCGA carrying an *ATM* TV [49]. However, the absence of HRD in *ATM*-associated tumours does not exclude the possibility that HetAT subjects who develop BC may be sensitive to cisplatin and/or poly(ADP)-ribose polymerase (PARP) inhibitors, as reported by others for HetAT subjects who developed prostate cancer [50]. Furthermore, it was shown that olaparib induces significant killing of *ATM*-deficient lymphoid tumour cells from patients with chronic lymphocytic leukaemia [51].

Interestingly, tumour genomic profiling revealed that ~ 70% of *ATM*-associated breast tumours are tetraploid. Polyploidy can be triggered by cell fusion, endoreplication or abortive cell cycle [52]. *ATM* is required for three cell cycle checkpoints- G_1_/S border, S phase and G_2_/M- after DNA double-strand breaks, so the emergence of polyploidy could be due to cell cycle checkpoint defects linked to inactivation of *ATM* in breast tumours. Of note, tetraploidy was also reported in *BRCA2-*associated tumours associated with the luminal molecular subtype and loss of the normal allele [53], although this result was not confirmed when *BRCA2* CNV profiles were investigated with SNP array and GAP methods [37].

We found that LOH at the *ATM* locus was more frequent in tumours from HetAT subjects (67%) than in ‘sporadic’ tumours from TCGA (40%) and from previous studies investigating LOH in tumours from sporadic BC cases (20–40%) [54, 55]. This observation is consistent with Knudson’s ‘two-hit’ model in which the second allele of the tumour suppressor gene would be an early event in the oncogenic process of hereditary BC. Similar results were found in *BRCA1-* and *BRCA2-*associated tumours [48]. With regard to *ATM*-associated tumours, one cannot exclude the possibility that biallelic inactivation of *ATM* occurs through promoter methylation of the gene in the seven tumours not showing LOH at the *ATM* locus or through point or small-size sequence variation (Fig. 1c). Another possible explanation for carriers of a deleterious missense variant would be that such alterations might have a dominant negative effect and therefore do not require inactivation of the second allele to impact gene product function. Finally, as previously reported, we did not observe a clear pattern of the biallelic inactivation of *ATM* according to variant type (TVs *vs*. deleterious or likely deleterious MS) [13].

In *ATM*-associated tumours, the cumulative profile of copy number losses, gains and regions in LOH revealed several genomic regions frequently altered in breast tumours, and in particular in luminal tumours, which was consistent with the molecular subtypes defined by IHC staining in our *ATM* series. Nevertheless, when comparing to TCGA sporadic tumours, the following copy number losses or regions of LOH appeared to be specific to *ATM*-associated tumours: 13q14.11-q14.3 (*LHFP*, *FOXO1*, *LCP1, RB1*), 21p11.2-p11.1 (*TPTE, TEKT4P2,* MIR3648-1, MIR3648-2, MIR3687-1, MIR3687-2) and 22q11.23 (*GSTT1, GSTTP1, GSTTP2*). Interestingly, loss of *RB1* has been associated with a poor response to hormone therapy [56], and expression of *LCP1* has been proposed as a biomarker of advanced tumour stage and tumour severity [57]. Unfortunately, in our study protein expression analysis could not be performed to validate the diminution of expression of these genes in *ATM*-associated tumours, owing to limited material.

To extend the repertoire of somatic alterations in *ATM*-associated tumours, we performed WGS on the four frozen tumours available. Only four deleterious variants located in the two genes *DNAH11* and *MUC4* were identified in two tumours. Little is known about the role of these two genes in tumourigenesis. However, the diminution of *MUC4* expression has been associated with tumour progression and with an increase infiltration of immune CD8+ T and natural killer cells [58]. Remarkably, no mutation in *TP53* and *PIK3CA* was detected in any of the four tumours, although these two genes are frequently mutated in luminal B tumours [59]. Despite the very limited sample size, we found that at the somatic level, *ATM*-associated tumours were more homogeneous in terms of CNV than in terms of SNV. In particular the two primary tumours from patient T0077 shared only 2.1% of SNVs. Moreover, no specific mutation signature as defined by Alexandrov *et al.* [60] could be identified using the SNV patterns of these four tumours only, and a larger tumour series should be sequenced to determine whether such signatures can discriminate *ATM*-associated tumours.

## Conclusions

Altogether, *ATM*-associated tumours do not show the hormone receptor deficiency profile, and it is not clear whether breast tumours developed by HetAT patients could be targeted by alkylating agents or PARP inhibitors [50, 51]. Nonetheless, hallmarks of *ATM*-associated tumours were found and could help to identify *ATM* variant carriers outside an A-T context or an HBOC family context. More studies are needed to investigate whether genes located at loci 13q, 21p and 22q could harbour potential new therapeutic targets and whether *RB1* deficiency could be a predictive biomarker for hormone therapy response for patients with BC carrying one or two mutated copies of *ATM*.

## Additional files

Additional file 1: **Table S1.** Genomic regions showing copy number loss or loss/LOH in at least 70% of *ATM*-associated tumours. (XLSX 70 kb)

Additional file 2: **Figure S1.** Copy number variation profiles of A*TM*-associated tumours analysed with the OncoScan array. **a** Genome-wide view of cumulative CNVs present in the 19 *ATM*-associated tumours from participants carrying one or two copies of a TV. Gains are indicated in *red*, losses in *blue*, and LOH in *orange*. **b** Cluster dendrogram and genomic regions altered in at least 70% of the 19 analysed tumours. AT children are indicated by asterisks. Loss: the two alleles are present in the tumour; Loss/LOH: only one allele is present in the tumour; Loss/LOH or Loss: consecutive segmental regions characterized as either ‘Loss/LOH’ or ‘Loss’. (PNG 510 kb)

Additional file 3: **Table S2.** Comparison of genes altered at the copy number level between *ATM*-associated tumours and tumours from TCGA. (XLSX 13 kb)

## Abbreviations

A-T: Ataxia-telangiectasia
*ATM*: Ataxia-telangiectasia mutated
BC: Breast cancer
*Bouin*: Bouin-fixed, paraffin-embedded tissue sample
CNV: Copy number variation
CoF-AT: French prospective cohort on women related to an A-T child
ER: Oestrogen receptor
*F*: Female
FET: Fisher’s exact test
*FFPE*: Formalin-fixed, paraffin-embedded tissue sample
GAP: Genome Alteration Print tool
HBOC: Hereditary breast and ovarian cancer
HER2: Human epidermal growth factor receptor 2
HES: Hematocylin and Eosin Stained
HetAT: Heterozygous *ATM* deleterious variant carrier
HRD: Homologous recombination deficiency
IHC: Immunohistochemistry
kConFab: Kathleen Cuningham Foundation Consortium for research into Familial Breast Cancer study
*L*: Left
LOH: Loss of heterozygosity
*M*: Male
MS: Missense substitution
Non-HetAT: Non-carrier of an *ATM* variant
PARP: Poly(ADP)-ribose polymerase
PICBIM: P*rogramme incitatif et collaboratif - Cancer du sein* series*: invasion et motilité*
PR: Progesterone receptor
*R*: Right
Retro-AT: French retrospective study on A-T families
SNP: Single-nucleotide polymorphism
SNV: Single-nucleotide variant
TCGA: The Cancer Genome Atlas
TNBC: Triple-negative breast cancer
*TV*: Truncating variant
WGS: Whole-genome sequencing
