# Supplementary figures and images for: Morphology and genomic hallmarks of breast tumours developed by ATM deleterious variant carriers
Source: Breast Cancer Res. 2018 Apr 17;20:28. doi: 10.1186/s13058-018-0951-9 (PMC5905168; doi:10.1186/s13058-018-0951-9)

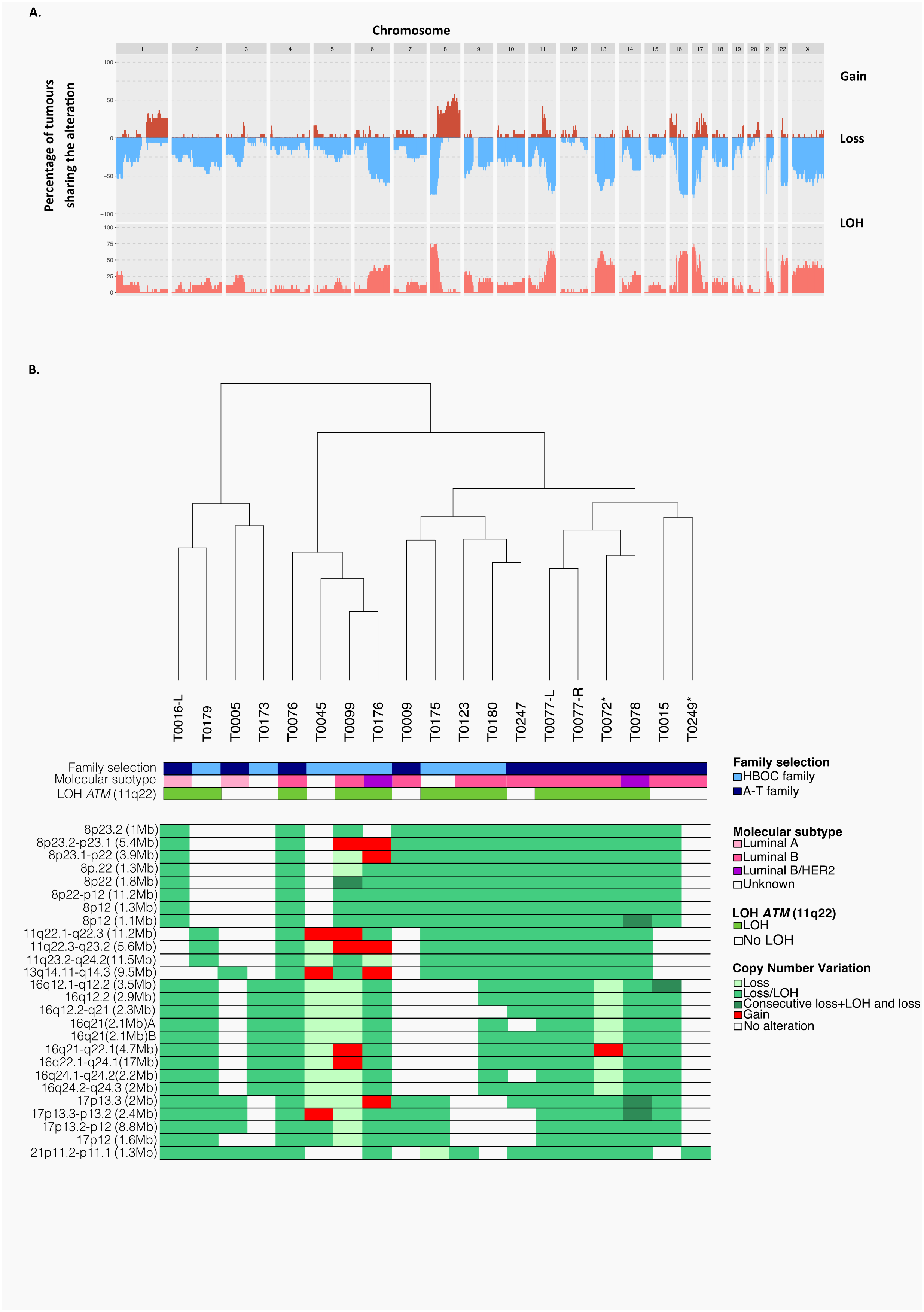

Supplement: Supplementary file 2 — Figure S1. Copy number variation profiles of ATM-associated tumours analysed with the OncoScan array. a Genome-wide view of cumulative CNVs present in the 19 ATM-associated tumours from participants carrying one or two copies of a TV. Gains are indicated in red, losses in blue, and LOH in orange. b Cluster dendrogram and genomic regions altered in at least 70% of the 19 analysed tumours. AT children are indicated by asterisks. Loss: the two alleles are present in the tumour; Loss/LOH: only one allele is present in the tumour; Loss/LOH or Loss: consecutive segmental regions characterized as either ‘Loss/LOH’ or ‘Loss’. (PNG 510 kb) [file 13058_2018_951_MOESM2_ESM.png]
